# Improvement of cardiac function by Ivabradine in a doxorubicin-induced cardiomyopathy murine model is associated with a normal renal angiotensin II type I receptor expression but not with a reduction in fibrosis

**DOI:** 10.1186/s40959-026-00469-z

**Published:** 2026-04-06

**Authors:** Gergana O. Drumeva, Anne-Flore Plane, Daniil R. Petrenyov, Paula A. B. Ribeiro, Cen Chen, Jean N. DaSilva, François B. Tournoux

**Affiliations:** 1 Centre de Recherche du Centre Hospitalier de l’Université de Montréal (CRCHUM), 900 St Denis Street, Montréal, Canada; 2 Département de pharmacologie et physiologie, Université de Montréal, Montréal, Canada; 3 Département de cardiologie, CHU de Caen, Caen, F-14032 France; 4 Département de radiologie, radio-oncologie et médecine nucléaire, Faculté de médecine, Université de Montréal, Montréal, Canada; 5 Service de cardiologie du Centre Hospitalier de l’Université de Montréal, Montréal, Canada

**Keywords:** Heart failure, Cardiac remodeling, Doxorubicin chemotherapy, Angiotensin II type 1 receptor, Medical care, Echocardiography, Autoradiography, Fibrosis

## Abstract

**Background:**

Ivabradine (IVAB) is an effective drug in patients with heart failure (HF). However, data in the context of chemotherapy-induced cardiomyopathy are limited. This study investigated the cardioprotective potential of IVAB in a doxorubicin (DOXO)-induced HF murine model, evaluating its effects on cardiac function, fibrosis, and its interaction with the renin-angiotensin system.

**Methods:**

C57BLC/6 female mice (*n* = 36) completed the protocol and were allocated into 2 groups: control (CTRL, *n* = 4) and treatment by DOXO (*n* = 32). DOXO administration (4 mg/kg/week, intraperitoneal) was performed over 5 weeks and followed by a 10-week gavage treatment with either water (H2O), IVAB (10 mg/kg/day), or metoprolol (METO, 100 mg/kg/day). Cardiac and kidney remodeling were assessed by echocardiography, pathology (with fibrosis assessed by picrosirius red (PSR) staining), and in vitro 125I-[Sar1,Ile8]Angiotensin II autoradiography for the measurement of AT1R levels.

**Results:**

After completion of DOXO injections, all mice demonstrated a lower cardiac function versus baseline (*p* < 0.0001). During therapy administration, only IVAB reduced heart rate (*p* < 0.0001) and improved cardiac function (*p* < 0.05). One week after the end of therapy , 1) cardiac function in IVAB group declined to levels similar to the other groups; 2) there was no difference in cardiac mass between groups, but fibrosis was increased in all DOXO-groups vs. CTRL (H2O +47%, *p* = 0.055; IVAB +90%, *p* < 0.001 and METO +47% in kidneys, *p* = 0.058); and 3) renal AT1R level was reduced only in the H2O group versus CTRL (-421%, *p* < 0.01), while IVAB and METO groups maintained renal levels comparable to controls.

**Conclusions:**

IVAB transiently improved systolic function in this model, but this benefit was not sustained after treatment cessation and did not prevent late structural fibrosis remodeling. Both IVAB and METO prevented the reduction of renal AT1R expression observed in DOXO treated animals. Overall, these findings indicate that HR-lowering therapy with IVAB alone is insufficient to prevent fibrotic remodeling, highlighting the need for longer-term studies to evaluate its sustained efficacy and impact on structural remodeling, and to assess its translational potential in managing DIC in patients.

**Graphical Abstract:**

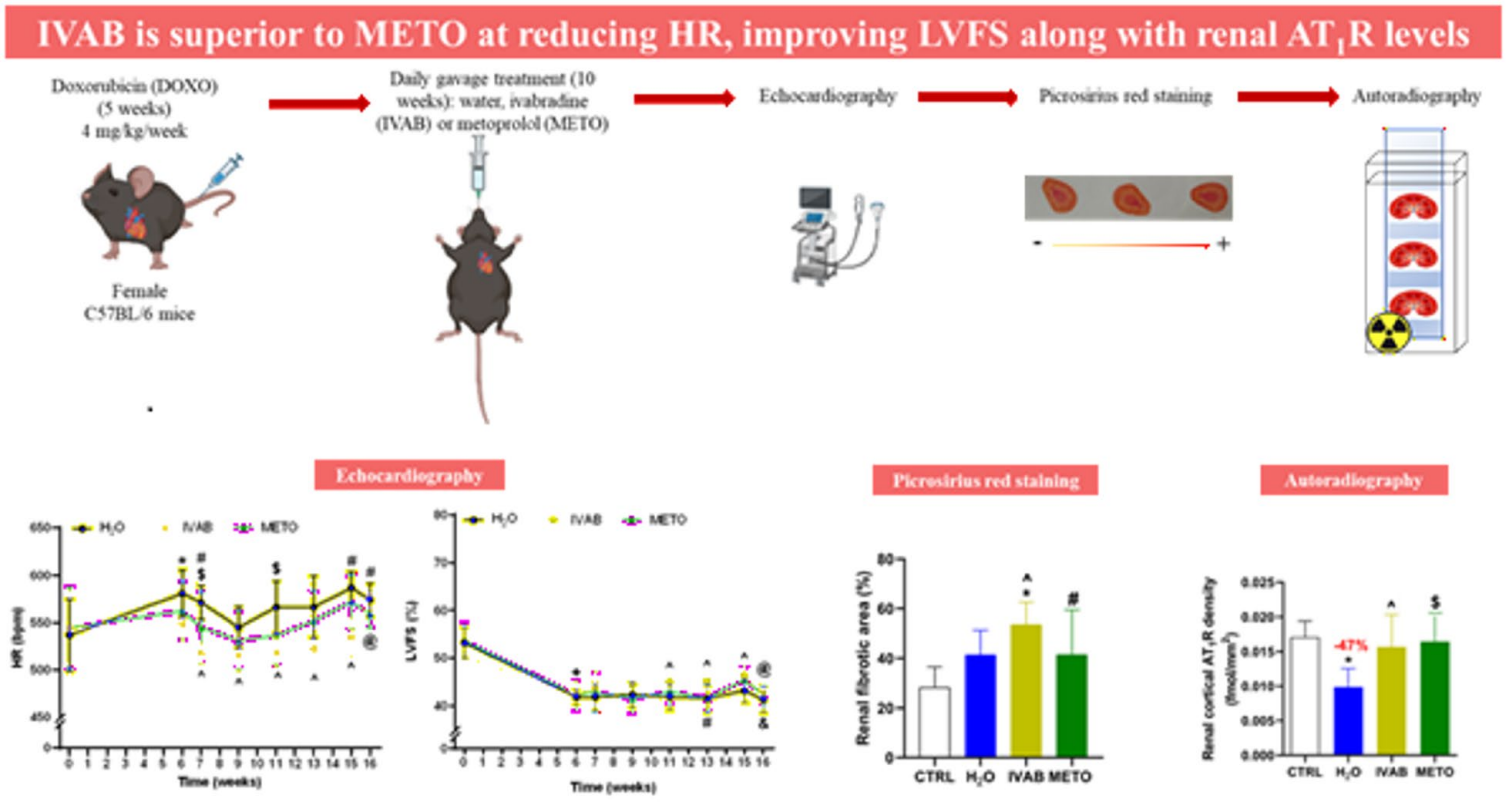

**Supplementary Information:**

The online version contains supplementary material available at 10.1186/s40959-026-00469-z.

## Introduction

Anthracyclines, such as doxorubicin (DOXO), are effective anti-cancer agents, but one of their main side effects is to induce cumulative and dose-dependent cardiotoxicity, which ranges from alterations in the myocardial structure and function to severe cardiomyopathy and congestive heart failure (HF) [1, 2]. This condition can affect up to 9% of patients with cancer who were treated with DOXO [3]. Suggested mechanisms of DOXO-induced cardiomyopathy (DIC) involve inflammation, apoptosis, mitochondrial dysfunction, calcium dysregulation, and oxidative stress [4]. There is increasing evidence that dysregulation of the renin-angiotensin system (RAS), particularly elevated levels of angiotensin II (Ang II) and altered Ang II type 1 receptor (AT_1_R) expression, may trigger DIC [5].

Heart rate (HR) is a major determinant of myocardial oxygen supply [6], and prolonged tachycardia is an important remodeling factor for the development of HF with reduced ejection fraction (HFrEF) [7, 8]. Beta-blockers (BB), such as metoprolol (METO), are effective in treating HF by selectively inhibiting β-1 adrenergic receptors, which reduces the patient’s HR [9]. BB could help prevent DIC and improve cardiac function [10, 11], although prior meta-analyses revealed inconsistent results [12]. BB may also cause significant side effects among these patients, such as low blood pressure, and contribute to overall significant fatigue and impaired quality of life.

As BB, *If*-channel blocker ivabradine (IVAB) significantly reduces HR via its negative chronotropic activity [7] but has no impact on patient’s blood pressure. IVAB has been proven to improve symptoms and reduce cardiovascular deaths and hospitalizations in HF patients [13]. It may offer benefits for patients with DIC, but the evidence in this specific context is still scarce.

The aims of this study were 1) To assess the effects of IVAB on structural and functional cardiac remodeling in a murine model of DOXO-induced cardiomyopathy, and 2) to investigate its potential interaction with the renin-angiotensin system, as the cardioprotective effects of IVAB may extend beyond HR reduction.

## Methods

### Study design

This is a 2-phase experimental animal study conducted over 16 weeks. Phase 1 consisted of active DOXO treatment for 5 weeks. Phase 2 involved the same DOXO-treated animals separated into 3 groups and focused on evaluating chronic effects and post-cardiotoxicity therapy over 10 weeks, during which the DOXO-treated groups received either water (H_2_O), IVAB or METO. In parallel to these 3 DOXO-treated groups, a 4th group (control) consisted in mice which did not receive DOXO or any treatment.

The specific objectives of this study were to evaluate, in a murine model of DIC, the impact of IVAB and METO on:

1. Cardiac structural and functional remodeling assessed by echocardiography: end-diastolic left ventricular (LV) posterior wall thickness (PWd) and interventricular septum (IVSd); LV end-diastolic diameter (LVEDd) and LV end-systolic diameter (LVEDs); and LV fractional shortening (LVFS).
2. Myocardial and renal fibrosis.
3. Cardiac and renal AT_1_R expression.

### Animals

**Fig. 1:**
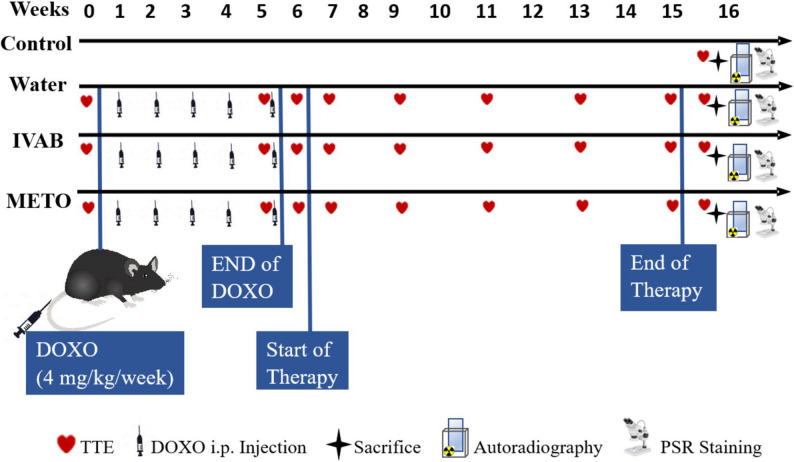
Schematic diagram of the study design. After baseline transthoracic echocardiography (ТТЕ), all animals except controls received weekly DOXO i.p. injections for 5 weeks, followed by 10 weeks of treatment with H_2_O, IVAB (10 mg/kg/day), or METO (100 mg/kg/day) by gavage. ТТЕ was performed at multiple timepoints, and at week-16, animals were sacrificed, and tissues were collected for autoradiographic studies and picrosirius red (PSR) staining. DOXO: Doxorubicin, IVAB: Ivabradine, METO: Metoprolol

Female C57BL/6 mice (6 weeks old; *n* = 40; Charles River Laboratories) were housed in a temperature-controlled facility on a 12:12 h light/dark cycle and fed standard mouse chow and water *ad libitium*. Anthracycline-induced heart toxicity varies by sex, affecting susceptibility, phenotype, and molecular pathways. Because we aimed to replicate anthracycline exposure in breast cancer (a condition primarily affecting women) this study exclusively used female mice. It is important to note that male mice tend to develop more severe cardiomyopathy and face higher mortality rates in chronic DOXO models [14, 15]. Mice were divided into two groups: a control group (CTRL) (*n* = 4) and an intervention group (*n* = 36). Starting at week-1, mice in the intervention group received intraperitoneal injections of DOXO (4 mg/kg/week) for 5 weeks and then randomized into three subgroups, receiving for 10 weeks by gavage either water (H_2_O, 0.1 mL; *n* = 13, representing no treatment group), or IVAB 10 mg/kg/day (*n* = 12), or METO 100 mg/kg/day (*n* = 11). The mice were weighed weekly, and echocardiography was performed at weeks: 0, 5, 6, 7, 9, 11, 13, 15 (at end of therapy), and 16 (one week after end of therapy). Mice were sacrificed at the end of the study (week-16) (Fig. 1) or when they showed signs of suffering (i.e., such as > 20% weight loss, visible signs of suffering, group isolation, or severe respiratory symptoms). CTRL mice were all scanned at week-16 (for age matching). Picrosirius red (PSR) staining and in vitro ^125^I-[Sar^1^,Ile^8^]Ang II autoradiography were performed on excised heart and kidney tissues (Fig. 1).

### Echocardiography

Transthoracic echocardiography (TTE) were performed using a Vivid 9 echocardiography machine and a 13-MHz probe (General Electric, USA). The examination was carried out under general anesthesia with isoflurane (2% at induction and 0.5-1% for maintenance). The mice were asleep in a box, shaved, and kept on a heating pad for the duration of the ultrasound testing. The parameters recorded were: HR, PWd, IVSd, LVEDd, LVEDs, and LVFS. For each examination, 3 acquisitions were realized, processed, and averaged. To be comparable, the difference in HR between exams had to be less than 100 bpm [16]; otherwise, measurements were excluded or repeated. To assess the effectiveness of the drugs, the HR was also measured with a PhysioSuite^®^ device, a sensor placed at the tail or the paw of the animal, after induction of isoflurane.

### Anesthesia and necropsy

Mice were weighed and anesthetized with 3% isoflurane: 0.5 L/min O_2_, after checking for the absence of reflex to pinching the hind legs. Euthanasia was carried out after opening the abdominal plane along the white line, by complete exsanguination via the inferior vena cava, with a 25G needle rinsed with 2% EDTA, and by a provoked pneumothorax. Prior to storing at -80 °C, the plasma was collected by centrifuging the whole blood at 1,000 x g for 5 min at 4 °C. Hearts and kidneys were excised, stripped of fat, rinsed in PBS, weighed, and immersed in O.C.T™ Compound (Sakura Finetek USA Inc., Torrance, California, USA), frozen on dry ice and stored at -80 °C.

### Histological assessments

Picrosirius red (PSR) staining was performed on tissue sections of all animals enrolled in the study. Twenty µm heart tissue sections were prepared at -20 °C with a Frigocut 2800 cryostat (Reichert-Jung/Leica, Wetzlar, Germany) and thaw-mounted on glass slides (Thermo Fischer Scientific, Waltham, Massachusetts, USA) and stored at -80 °C. On the day of the histological analysis, slides were left at room temperature for a few minutes. Heart and kidney slides were rehydrated with 100-80-40% ethanol (2 min each) and washed with deionized water for 10 s. Following 10% formalin (Thermo Fischer Scientific, Waltham, Massachusetts, USA) incubation for 30 min, slides were washed with deionized water (2 × 5 min) and incubated with PSR staining (Thermo Fischer Scientific, Waltham, Massachusetts, USA) for 2 h. Slides were then washed in acidified water (0.5% acetic acid, Sigma-Aldrich, St. Louis, Missouri, USA) and deionized water (2 × 2 min), and dehydrated with 2 changes of 95% ethanol and 100% ethanol. They were finally cleared in 2 changes of xylene (Thermo Fischer Scientific, Waltham, Massachusetts, USA) for 2 min and mounted in a resinous medium VectaMount (Vector Laboratories Inc., Burlingame, California, USA). PSR-stained specimens were imaged at ×16 magnification with a ZEISS Stemi 508 bright field microscope (ZEISS, Oberkochen, Germany) equipped with an Axiocam 105 camera (ZEISS, Oberkochen, Germany) and analyzed with ImageJ 1.54f software (NIH, USA). Specifically, red-stained pixels (identifying fibrosis tissue) were quantified and calculated as a percentage of all pixels (fibrosis, whole myocardium, kidney), as previously described [17, 18].

### In vitro autoradiography

 In vitro ^125^I- [Sar^1^, Ile^8^] Ang II autoradiography was carried out using the methods published previously [19]. ^125^I- [Sar^1^, Ile^8^] Ang II (Revvity, Waltham, Massachusetts, USA) was supplied as a powder that was freshly dissolved in distilled water, and each vial contains 10 µCi (0.37 MBq) with a specific activity of 2200 Ci (81.4 TBq)/mmol. Organs of interest were sectioned as follows: 20 μm-thick kidney and heart sections prepared at -20 °C with Frigocut 2800 cryostat (Reichert-Jung/Leica, Wetzlar, Germany). Tissue sections were thaw-mounted onto glass slides (Thermo Fischer Scientific, Waltham, Massachusetts, USA) and stored at -80 °C. On the day of the experiment, slides were preincubated in assay buffer (150 mM NaCl (Thermo Fischer Scientific, Waltham, Massachusetts, USA), 50 mM Na_2_HPO_4_ dibasic (VWR, Radnor, Pennsylvania, USA), 1 mM EDTA (Wisent Inc., Saint-Jean-Baptiste, Québec, Canada), 0.1 mM bacitracin (Thermo Fischer Scientific, Waltham, Massachusetts, USA) and 0.1% BSA (pH 7.4 ,VWR, Radnor, Pennsylvania, USA) for 15 min at room temperature, in the presence of the Ang II type 2 receptor (AT_2_R) antagonist PD 123, 319 (10 µM, Alomone Labs, Jerusalem, Israel) or with unlabeled Ang II (10 µM, Alomone Labs, Jerusalem, Israel) to determine total non-AT_2_R binding (TB) or non-specific binding (NSB), respectively. The slices were then incubated with 0.25 nM of ^125^I- [Sar^1^, Ile^8^] Ang II for 90 min at room temperature. Following incubation, the slides were sequentially washed with deionized water (2x), buffer (4x, 2 min), and deionized water (2x) at 4 °C, and then air-dried. Heart sections were exposed along with a set of radioactivity standards onto an imaging phosphor screen (Cytiva, Marlborough, Massachusetts, USA) for 5 days, whereas kidney sections were exposed for 2 days. The screens were imaged at 50 μm resolution with a molecular imager Typhoon Trio (Cytiva, Marlborough, Massachusetts, USA) and analyzed using ImageJ 1.53k software (NIH, USA). Quantification of the samples was performed by manually tracing the kidney cortex and the heart’s contour and the corresponding ^125^I- [Sar^1^, Ile^8^] Ang II uptake (fmol/mm^2^) was calculated for that area. The AT_1_R specific binding (SB) is calculated as TB minus NSB.

### Statistical analysis

Data were expressed as means ± standard deviation (SD). Baseline differences for mice weight, HR, LVEDd, LVEDs, IVSd, PWd, and LVFS for all animals were tested using a paired T-test (week-0 vs. week-6). Groups’ differences were tested using One-Way ANOVA with post-hoc (Tukey’s Multiple Comparison) at 6, 7, 9, 11, 13, 15, and 16 weeks. Group differences for AT_1_R expression and fibrosis at week-16 were tested using One-Way ANOVA with post-hoc (Tukey’s Multiple Comparison). Statistical analyses were performed with GraphPad Prism Version 8. 1. 0. A significant difference was defined as *p* ≤ 0.05.

## Results

Four mice were excluded from the final analysis: two mice from IVAB group and two mice from METO group died a few weeks post-DOXO injection. Therefore, *n* = 36 mice were included in the final analyses (CTRL *n* = 4; H_2_O *n* = 13; IVAB *n* = 10; METO *n* = 9).

Mice in the H_2_O group exhibited an 11% increase in body weight at the end of DOXO treatment (week-0 vs. week-6; *p* < 0.0001; Fig. S1A). Mice in the IVAB group displayed 9% body weight increase (*p* < 0.01) and METO group exhibited a 14% body weight increase (*p* < 0.001) following DOXO treatment. Throughout the rest of the study, body weight remained stable across all animal groups (Fig. S1A).

### IVAB significantly reduced HR, not METO

**Fig. 2:**
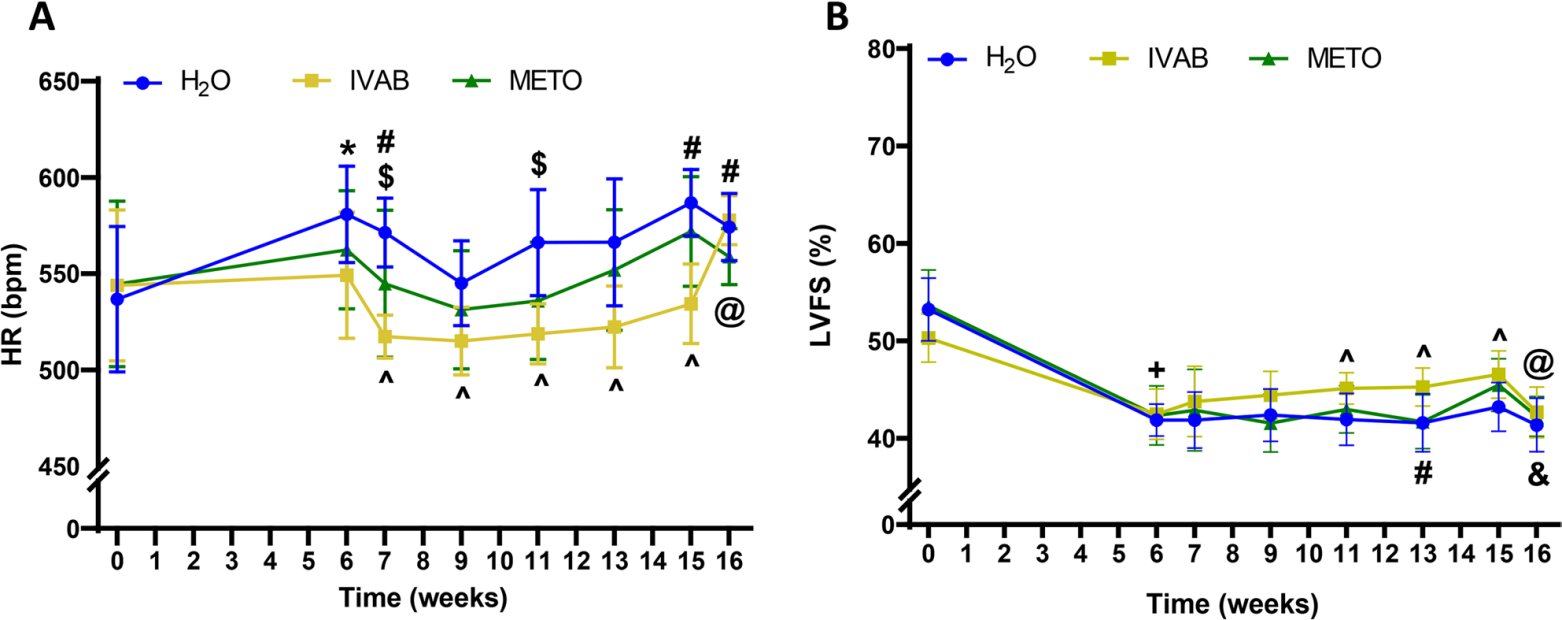
Effects of DOXO, IVAB and METO on HR and LVFS. Transthoracic echocardiography was performed at each timepoint to assess HR (**A**) and LVFS (**B**) in all animal groups. DOXO: Doxorubicin, IVAB: Ivabradine, METO: Metoprolol, HR: Heart rate, LVFS: left ventricle fractional shortening. Data are presented as means ± SD. *p < 0.05 H_2_O Week-0 vs. Week**-**6, +*p* < 0.0001 Week-0 vs. Week**-**6 for H_2_O, IVAB, and METO, ^*p* < 0.05 H_2_O vs. IVAB, #*p* < 0.05 IVAB vs. METO, $*p* < 0.05 H_2_O vs. METO, &*p* < 0.05 METO Week-15 vs. Week-16; @*p* < 0.01 IVAB Week-15 vs. Week-16

After 5 weeks of DOXO (at week-6), there was a significant increase in HR in H_2_O group when compared to baseline: 536.7 ± 37.8 bpm at baseline vs. 580.8 ± 25.1 bpm (*p* < 0.05, Fig. 2A, Table S1). Animals in IVAB and METO group, which had not yet started HF therapy, did not exhibit a change in HR following DOXO. Throughout the intervention period (i.e., therapy administration), IVAB consistently reduced HR to a larger extent than METO (Fig. 2A). After 10 weeks of therapy (week-15), HR observed within the IVAB group was significantly lower than that of the H_2_O group (534 ± 21 bpm vs. 587 ± 17 bpm, (*p* < 0.0001, Table S1). However, one week after the cessation of IVAB (week-16), HR increased to 578 ± 13 bpm compared to week-15 (*p* < 0.001), reaching a similar level to that of H_2_O mice (574 ± 17 bpm, Table S1). After 10 weeks of therapy, METO had no significant effect on HR, nor was there any change one week after cessation, compared to H_2_O group. For CTRL animals, HR was 500.8 ± 34.6 bpm at week-16.

### LVFS was rapidly improved by IVAB, but declined after discontinuation

After 5 weeks of chemotherapy with DOXO, there was a significant reduction in LVFS when compared to baseline in all animals: for H_2_O: 53.2 ± 3.2% at baseline vs. 41.9 ± 1.6%; for IVAB: 50.3 ± 2.5% at baseline vs. 42.5 ± 2.6%; for METO: 53.6 ± 3.7% at baseline vs. 42.3 ± 3.0% (*p* < 0.0001 for all, Fig. 2B, Table S1). Following the initiation of therapy (H_2_O, IVAB, or METO) at week-6, an improvement in LVFS was observed exclusively with IVAB during the follow-up period. In the IVAB group, LVFS significantly increased by week-11 and continued to improve over time, reaching 46.5 ± 2.4% at week-15 (*p* < 0.05, Fig. 2B). METO did not improve LVFS compared to H_2_O group over time. One week after IVAB being stopped (week-16), cardiac function quickly declined, with an LVFS of 42.7 ± 2.6% (*p* < 0.01, Table S1), compared to week-15. Increases in LV cavity sizes were observed after DOXO treatment at week-6 for all animals (LVEDd: *p* < 0.001 for H_2_O, *p* < 0.01 for IVAB and METO; LVEDs: *p* < 0.0001 for H_2_O and METO, *p* < 0.001 for IVAB, Table S1) compared to week-0. An increase in IVSd was observed following DOXO (*p* < 0.05) compared to baseline, whereas no change has been detected in PWd (Table S1). No change in LV cavity sizes was observed at the end of therapy (week-15) as compared to H_2_O group. For CTRL animals, LVFS was 55.3 ± 2.6% at week-16 with normal LV size (LVEDd 3.4 ± 0.2 mm). No significant differences were observed in heart-to-body weight ratios (Fig. S1B).

### HF therapies led to significant fibrotic changes

**Fig. 3:**
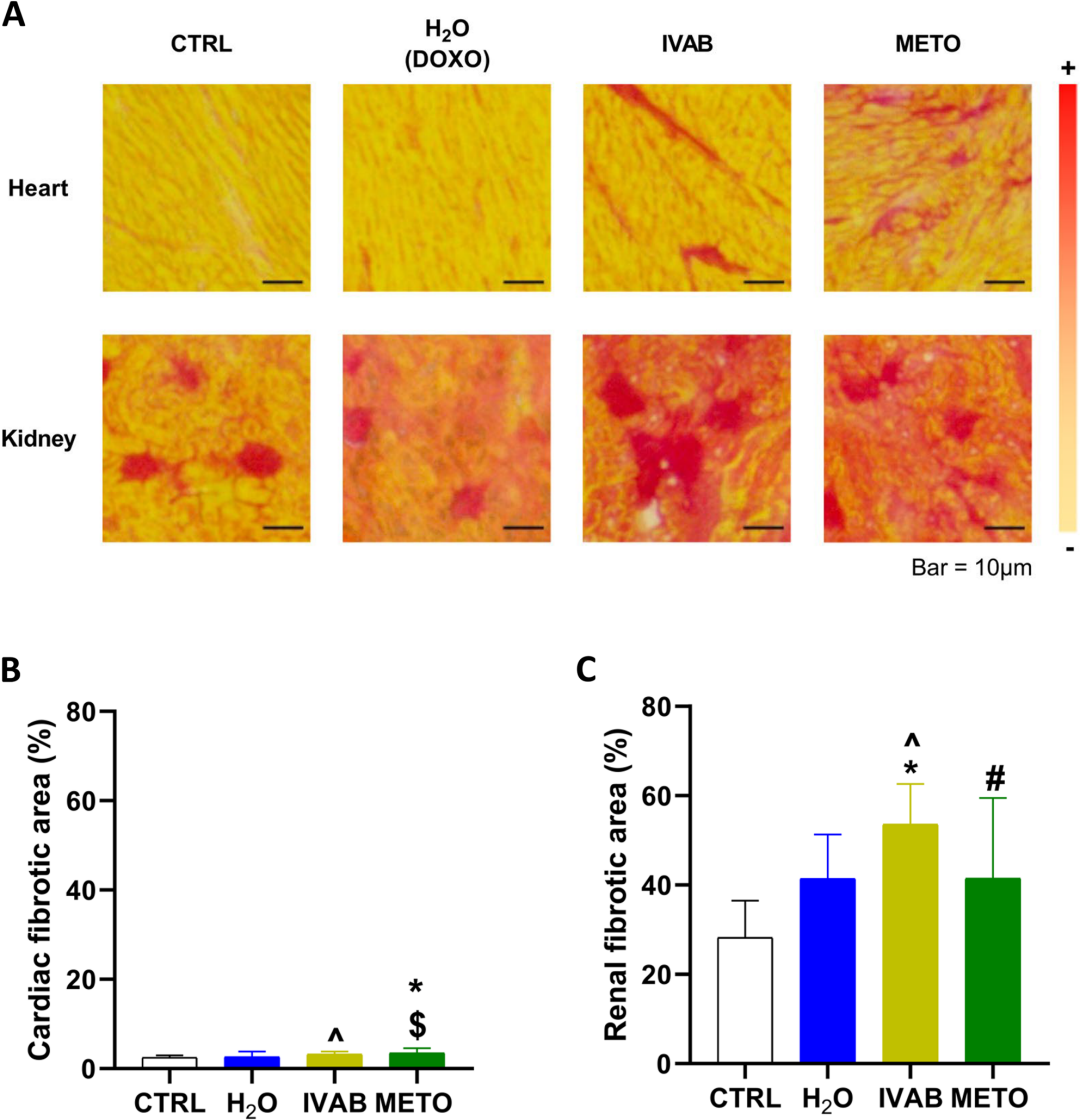
Structural remodeling in the heart and kidneys following DOXO, IVAB and METO. **A** Representative images of picrosirius red staining in the heart and kidneys. Elevation of fibrosis towards the « + ». Quantification of fibrosis in the (**B**) heart and (**C**) kidneys. DOXO: Doxorubicin, IVAB: Ivabradine, METO: Metoprolol. Data are presented as means ± SD. *p < 0.05 vs. CTRL, ^*p* < 0.05 H_2_O vs. IVAB, #*p* < 0.05 IVAB vs. METO, $*p* < 0.05 H_2_O vs. METO

Administration of DOXO over 5 weeks was not associated with significant changes in intracardiac fibrotic areas (2.6 ± 1.2% in H_2_O group vs. 2.5 ± 0.5% in CTRL, *p* = 0.98, Fig. 3A and B). However, there was a significant increase in fibrotic areas in the hearts of METO group when compared to CTRL animals (3.5 ± 1.1%, *p* < 0.05) and to H_2_O groups (*p* < 0.01). There was also an increase in fibrosis in the hearts of IVAB groups when compared to H_2_O animals (3.3 ± 0.6%, *p* < 0.05). In the kidney, there was an increase in fibrotic areas following DOXO administration (28.3 ± 8.2 in CTRL vs. 41.4 ± 9.9% in H_2_O group; p value = 0.055, Fig. 3A and C). Kidneys from the IVAB and METO groups also displayed an increased fibrosis, when compared to CTRL (53.6 ± 9.1%, *p* < 0.001; 41.5 ± 18.1%, p value = 0.058, respectively), whereas only the IVAB group showed an increased fibrosis compared to H_2_O (*p* < 0.05). There was also a renal fibrotic difference between IVAB and METO groups (*p* < 0.05). No significant differences were observed in kidney-to-body weight ratios (Fig. S1C).

### Renal AT_1_R expression was reduced only in H_2_O group

**Fig. 4:**
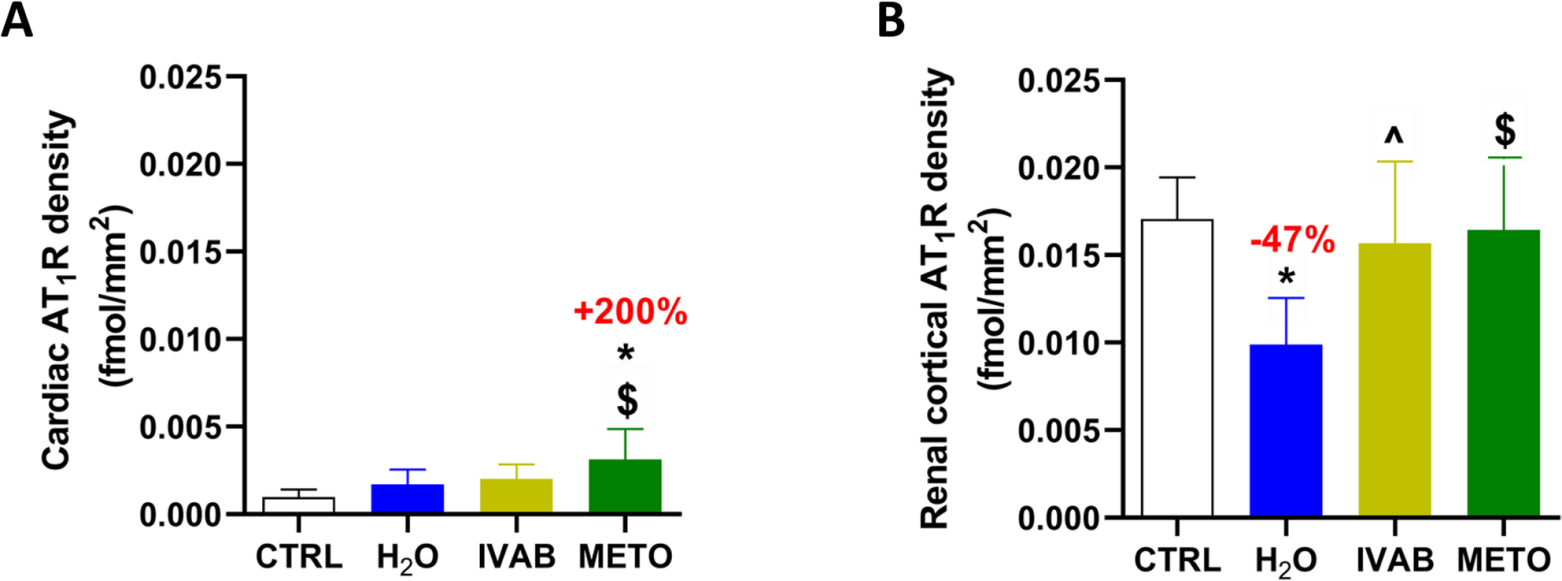
AT_1_R density in the (**A**) heart and (**B**) kidneys at the end of the study. AT_1_R: Angiotensin II type 1 receptor, IVAB: Ivabradine, METO: Metoprolol. Data are presented as means ± SD. *p < 0.05 vs. CTRL, ^*p* < 0.05 H_2_O vs. IVAB, $*p* < 0.05 H_2_O vs. METO

Cardiac AT_1_R density was 0.001 ± 0.001 fmol/mm^2^ in CTRL animals. Administration of DOXO not followed by any HF therapy did not affect cardiac AT_1_R density at week-16 compared to control mice (0.002 ± 0.001 fmol/mm^2^ in H_2_O group, see Fig. 4A). At week-16 (5 weeks DOXO and 10 weeks of treatment), METO animals showed a 200% increase of cardiac AT_1_Rs to 0.003 ± 0.002 fmol/mm^2^ compared to control and H_2_O animals (*p* < 0.05), whereas IVAB showed no effect on cardiac AT_1_R density at week-16.

In the kidney cortex of control animals, AT_1_R density was 15–20 times higher than within the myocardium (0.017 ± 0.002 fmol/mm^2^). Administration of DOXO not followed by any HF therapy was associated with a 47% significant decrease in AT_1_R density at week-16 compared to CTRL mice (0.009 ± 0.003 fmol/mm^2^ in H_2_O animals; *p* < 0.01). IVAB and METO treatments demonstrated renal AT_1_R expression of 0.016 ± 0.004 fmol/mm^2^ and 0.019 ± 0.002 fmol/mm^2^ (*p* = 0.97), respectively, similar to the value observed in CTRL animals (Fig. 4B).

## Discussion

DIC continues to present challenges in clinical practice [20] and has limited the use of anthracyclines for treating leukemia, lymphomas, and a number of solid tumors [21]. In this murine DIC model evaluating the effects of IVAB and METO, we observed that 1) only IVAB significantly improved cardiac function (however, one-week post-treatment cardiac function in the IVAB group declined to levels comparable with the other groups); 2) cardiac and renal fibrosis increased in only DOXO animals treated by either IVAB or METO; and 3) renal AT_1_R expression level was only reduced in the H_2_O group.

In accordance with previous studies [16, 22], we observed a significant decrease of 8–11% of LVFS in our models after cumulative doses of DOXO injection. This reduction in cardiac function was sustained over the entire study follow-up. In animals treated with METO or IVAB, cardiac function improved over time in IVAB animals only. Such improvement by IVAB has been previously reported in both animal and clinical studies. IVAB effectively reduced HR and improved diastolic and systolic LV function in a male rat model of DIC [23], and significantly improved cardiac function and alleviated symptoms in patients with cancer treatment-related LV dysfunction [24]. Anti-inflammatory and anti-oxidant properties such as the inhibition of vascular lipid peroxidation, vascular superoxide production, and vascular Nicotinamide Adenine Dinucleotide Phosphate (NADPH) oxidase activity [25, 26] have been suggested to explain the cardioprotective effect of IVAB, but the exact mechanisms are still unknown.

In our animal model, improvement in cardiac function by IVAB was not associated with a reduction in fibrosis at week-16. The effect of this drug on myocardial fibrosis in various cardiovascular disease models is controversial, some demonstrating decreased fibrosis [27] (through reduced proliferation and activation of cardiac fibroblasts via c-Jun N-terminal kinase (JNK) and p38 mitogen-activated protein kinase (p38 MAPK) inhibition [28]), while others reported no difference [29]. For METO, previous studies did not show any prevention of cardiac fibrosis in DOXO-treated mice [30] by this drug. METO and DOXO administration significantly reduces the cardiac expression of sirtuin-3 (SIRT3) mRNA, a key metabolic sensor in the heart [30], which alleviates cardiac hypertrophy and fibrosis [31]. Its blockade leads to a decrease of the protective SIRT3 pathway against oxidative stress, mitochondrial function, cardiac fibrosis, and cardiac function, especially in cancer treatment-related cardiomyopathy [30].

Given the limited impact of IVAB and METO on myocardial fibrosis despite cardiac function improvements, we next sought to determine whether these treatments - neither of which directly targets the RAS - could nonetheless influence molecular remodeling pathways, specifically the expression of AT_1_R. Due to the low cardiac AT_1_R expression, the important heart-kidney interaction, and the high abundance of AT_1_R in the kidneys, we assessed this expression in both organs. To the best of our knowledge, this was the first study to assess the potential impact at the AT_1_R level of two well-established HF therapies (IVAB and METO) on both heart and kidney in a murine model of DIC. In the present study, AT_1_R expression was significantly reduced in response to DOXO, which may be due to an increase in plasma Ang II levels, as there is a negative feedback regulation between Ang II and AT_1_R [19, 32]. Cardiac AT_1_R expression in rats is around 40-fold lower than that in the kidneys, which aligns with our results displaying lower cardiac AT_1_R levels in mice, as characterized by autoradiography [33], and consistent with other findings using positron emission tomography (PET) imaging [34]. Interestingly, IVAB and METO prevented the reduction of renal AT_1_R expression observed in DOXO-treated animals, resulting in expression levels comparable to those of control animals, which is in line with previous studies establishing the pleiotropic effects of IVAB, besides its HR reduction [35]. IVAB reduces protein and gene expression of LV angiotensin converting enzyme (ACE) and AT_1_R [36, 37], with its protective effect linked to a reduction in the aldosterone level and aldosterone/Ang II ratio, while serum renin remains unaffected [38]. METO’s impact on RAS is explained by a direct suppression of renin release [39] and plasma renin activity [40].

It can be inferred that the positive indirect impact of IVAB and METO on RAS was likely insufficient to impede the progression of fibrosis in this cardiomyopathy, which is considered as one of the most severe among non-ischemic causes of HF [41]. This observation may also clarify why, upon cessation of IVAB, a recurrence of the cardiomyopathy was noted, manifested as a further decline in cardiac function. Additionally, Ang II acting through AT_1_R was reported to directly induce profibrotic signaling in human cardiac fibroblasts (e.g., increased TGF-β1 and extracellular gene expression), emphasizing that tissue-level signaling, rather than renal receptor abundance alone, determines myocardial fibrosis [42]. The beneficial effects of IVAB on LV function in this study are of slow onset, as they depend on chronic hemodynamic optimization and improved myocardial perfusion - processes that require time due to the gradual nature of ventricular remodeling and adaptation [43, 44]. In contrast, these benefits are rapidly lost after treatment interruption, since they are both dose- and HR–dependent [45], and an increase in HR quickly disrupts the hemodynamic balance, leading to a reversal of functional benefits. This may also explain why treating DIC with BBs alone [30] remains controversial, unlike with an ACE inhibitor (ACEi), for example, for which protective effects have been demonstrated [46, 47]. In humans, HF treatment is based on the use of all the drug classes recommended by guidelines [13], with each targeting a specific system involved in the progression of HF. However, it also raises concerns about patients who cannot tolerate all recommended HF medications. This could lead to the unintended overstimulation of unblocked systems by the medications they can tolerate. For example, sacubitril, which targets the natriuretic peptides system, does not show benefits for HF patients if not prescribed alongside an angiotensin receptor blocker [48–50]. Therefore, some of our results should be further investigated, as they may influence medication prescriptions in clinical practice.

Future research should focus on longitudinal assessment of AT_1_R expression to track dynamic changes immediately post-DOXO and during early recovery. Future investigations could measure AT_1_Rs levels at the end of treatment and 2–4 weeks post-treatment, since the animals were sacrificed only at the end of the study. Additional treatments to consider include ACEi, ACEi+METO, and ACEi+IVAB. To provide mechanistic insight, future studies could also include measurements of circulating and tissue levels of Ang I, Ang II, as well as aldosterone, and quantify cardiac downstream profibrotic signaling sites, such as Transforming Growth Factor-beta (TGF-β), Sma- and Mad-related protein 2/3 (Smad2/3) phosphorylation, and fibrosis-related gene expression in the LV. These analyses would allow to determine whether LV fibrosis in the IVAB- and METO-treated groups is associated or not with persistent local cardiac profibrotic signalling despite renal AT_1_R expression changes. Furthermore, longitudinal noninvasive assessment of AT_1_R expression could be even facilitated by PET imaging, using our newly developed tracers [51–53].

### Limitations

Recent reports have drawn attention to DOXO-induced cardiac atrophy in both mice and humans [54, 55], further highlighting the detrimental effects on heart function. However, we did not observe cardiac atrophy at the end of our study, which may indicate that the duration or dosage of DOXO treatment in our experiment was not sufficient to trigger a noticeable effect. Also, we examined AT_1_R expressions only at the end of the study. It would have been valuable to assess AT_1_R expression at other timepoints as well (e.g. just after completion of DOXO), and determine if the reduction observed at week-16 also occurs at the end of DOXO treatment. The concentration of treatment drugs was based on previous studies [56, 57]; however, using doses of IVAB and METO that produce comparable HR reduction would improve the interpretation of their differential effects. In addition, this study was conducted only in female mice, and potential sex-dependent effects in males were not assessed.

## Conclusions

This study offers observations on the effects of IVAB and METO on cardiac function and RAS in a murine model of DIC. Even though IVAB was the only drug associated with a transient improvement in LVFS, both IVAB and METO were associated with renal AT_1_R levels comparable to control values at the end of the study. However, the improvement in cardiac function was not sustained, as cardiac function declined after treatment cessation, and fibrosis progressed across all groups. Although HR-lowering IVAB therapy temporally improved cardiac function, it did not prevent late structural fibrosis in this model, even though renal AT1R levels were similar to those of the controls. Our results justify further testing, including postmortem AT_1_R assessments at multiple time points, which aligns with the need for novel imaging techniques for DIC. In the longer term, a more personalized approach may be considered when translating these results to clinical settings, considering the limited impact of some therapies on fibrosis. Additional research on targeted therapeutic combinations and molecular markers, such as AT_1_R, may help refine future treatment strategies.

## Supplementary Information

Supplementary Material 1.

## Data Availability

The data that support the findings of the current study are available from the corresponding author upon reasonable request.

## Abbreviations

ACE: Angiotensin converting enzyme
ACEi: ACE inhibitor
Ang II: Angiotensin II
AT_1_R: Angiotensin II Type 1 Receptor
AT_2_R: Angiotensin II Type 2 Receptor
BB: Beta-blockers
C57BL/6: C57BL/6 mouse strain
CTRL: Control
DIC: DOXO-induced cardiomyopathy
DOXO: Doxorubicin
HF: Heart Failure
H2O: Water
HFrEF: HF with reduced Ejection Fraction
HR: Heart rate
IVAB: Ivabradine
IVSd: Interventricular septum at end-diastole
JNK: c-Jun N-terminal kinase
LV: Left ventricular
LVEDd: LV end-diastolic diameter
LVEDs: LV end-systolic diameter
LVFS: LV fractional shortening
METO: Metoprolol
NADPH: Nicotinamide Adenine Dinucleotide Phosphate
NSB: Non-specific binding
O₂: Oxygen
p38 MAPK: p38 mitogen-activated protein kinase
PET: Positron emission tomography
PSR: Picrosirius red
PWd: LV posterior wall thickness
RAS: Renin-Angiotensin System
SB: Specific binding
SD: Standard deviation
SIRT3: Sirtuin-3
Smad2/3: Sma- and Mad-related protein 2/3
TB: Total non-AT_2_R binding
TGF-β1: Transforming Growth Factor-beta
TTE: Transthoracic echocardiography
