# Supplementary material for: Improvement of cardiac function by Ivabradine in a doxorubicin-induced cardiomyopathy murine model is associated with a normal renal angiotensin II type I receptor expression but not with a reduction in fibrosis
Source: Cardiooncology. 2026 Apr 6;12:59. doi: 10.1186/s40959-026-00469-z (PMC13173794; doi:10.1186/s40959-026-00469-z)
Supplement: Supplementary file 1 — Supplementary Material 1. [file 40959_2026_469_MOESM1_ESM.docx]

**Supplementary Appendix**


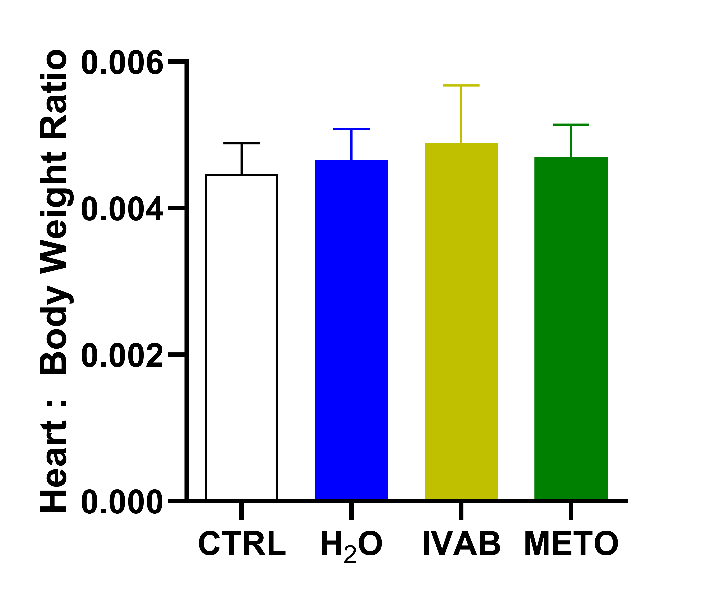


**B**


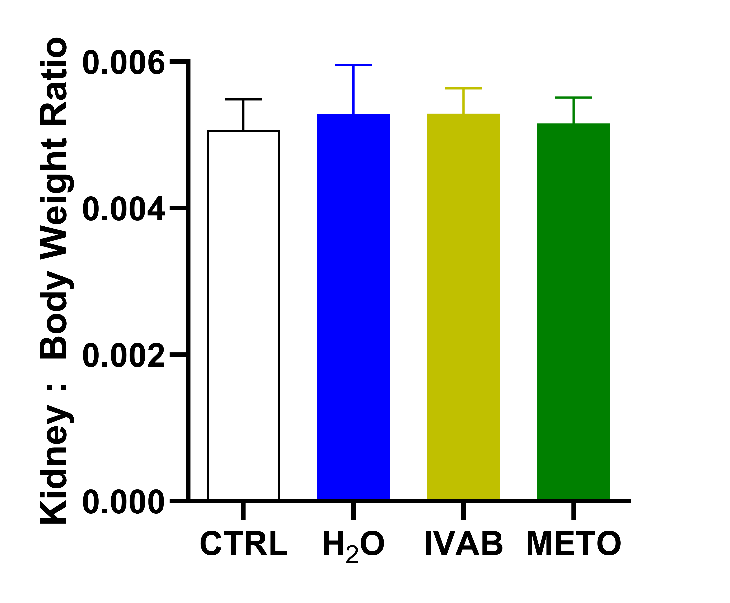


**C**

**A**

**Fig. S1. Body and organ weights. (A)** Animal weights throughout the study. **(B)** Terminal heart weight normalized to body weight. **(C)** Terminal kidney weight normalized to body weight. IVAB: Ivabradine, METO: Metoprolol. Data are presented as means ± SD. *p < 0.0001 Week-0 vs. Week**-**6 for H_2_O; ^p < 0.01 Week-0 vs. Week**-**6 for IVAB; #p < 0.001 Week-0 vs. Week**-**6 for METO.

**Table S1: Temporal echocardiographic parameters**

|  | Group | Baseline (Week-0) (means ± SD) | End of DOXO/Beginning of therapy (Week-6) (means ± SD) | End of therapy (Week-15) (means ± SD) | At sacrifice (Week-16)  (means ± SD) |
| --- | --- | --- | --- | --- | --- |
| Weight,  g | CTRL  H_2_O  IVAB  METO | 18.0 ± 1.5  18.2 ± 1.0  17.9 ± 1.3 | 19.9 ± 1.0^+^  19.9 ± 1.5**°**  20.4 ± 1.4^×^ | 21.4 ± 1.6  21.8 ± 2.0  21.8 ± 1.6 | 22.0 ± 1.8  21.4 ± 1.8  21.7 ± 2.4  22.2 ±1.8 |
| HR,  bpm | CTRL  H_2_O  IVAB  METO | 536.7 ± 37.8  543.9 ± 39.2  544.7 ± 43.0 | 580.8 ± 25.1*  549.2 ± 32.7  562.4 ± 30.7 | 586.9 ± 17.2  534.4 ± 20.7^  572.0 ± 28.4^#^ | 500.8 ± 34.6  574.3 ± 17.4  577.9 ± 12.7^@^  558.9 ± 14.5^#^ |
| LVEDd, mm | CTRL  H_2_O  IVAB  METO | 3.4 ± 0.2  3.4 ± 0.2  3.4 ± 0.2 | 3.6 ± 0.2^×^  3.7 ± 0.3**°**  3.6 ± 0.2**°** | 3.7 ± 0.1  3.7 ± 0.2  3.6 ± 0.2^#^ | 3.4 ± 0.2  3.8 ± 0.1  3.7 ± 0.2  3.7 ± 0.2 |
| LVEDs,  mm | CTRL  H_2_O  IVAB  METO | 1.6 ± 0.2  1.7 ± 0.2  1.6 ± 0.2 | 2.1 ± 0.2^+^  2.1 ± 0.2^×^  2.1 ± 0.2^+^ | 2.1 ± 0.1  2.0 ± 0.2  1.9 ± 0.2^$^ | 1.5 ± 0.1  2.2 ± 0.1^◊^  2.1 ± 0.2  2.1 ± 0.1^&^ |
| IVSd,  mm | CTRL  H_2_O  IVAB  METO | 0.7 ± 0.1  0.7 ± 0.1  0.7 ± 0.1 | 0.6 ± 0.04*  0.6 ± 0.1  0.6 ± 0.1 | 0.7 ± 0.1  0.7 ± 0.1  0.7 ± 0.1 | 0.8 ± 0.1  0.7 ± 0.1  0.7 ± 0.03  0.7 ± 0.0 |
| PWd,  mm | CTRL  H_2_O  IVAB  METO | 0.7 ± 0.1  0.6 ± 0.04  0.6 ± 0.1 | 0.6 ± 0.1  0.6 ± 0.03  0.6 ± 0.04 | 0.6 ± 0.03  0.6 ± 0.04  0.6 ± 0.03 | 0.8 ± 0.2  0.6 ± 0.04  0.6 ± 0.03  0.1 ±0.03 |
| LVFS,  % | CTRL  H_2_O  IVAB  METO | 53.2 ± 3.2  50.3 ± 2.5  53.6 ± 3.7 | 41.9 ± 1.6^+^  42.5 ± 2.6^+^  42.3 ± 3.0^+^ | 43.2 ± 2.5  46.5 ± 2.4^  45.5 ± 2.7 | 55.3 ± 2.6  41.3 ±2.8  42.7 ± 2.6^@^  42.2 ± 2.1^&^ |

CTRL group (n = 4), H_2_O (n = 13), IVAB (n = 10) and METO (n = 9). DOXO: Doxorubicin, IVAB: Ivabradine, METO: Metoprolol. SD: standard deviation, g: gram, HR: heart rate, bpm: beats per minute, LVEDd: left ventricular end-diastolic diameter, mm: millimeter, LVEDs: LV end-systolic diameter, PWd: posterior wall diameter, IVSd: intraventricular septal thickness in diastole, LVFS: left ventricle fractional shortening. Data are presented as mean ± SD. For Week-0 vs. Week-6: *p < 0.05, °p < 0.01, ×p < 0.001, +p < 0.0001; ^p < 0.05 H_2_O vs. IVAB, $p < 0.05 H_2_O vs. METO, #p < 0.05 IVAB vs. METO, ◊p < 0.05 H_2_O Week-15 vs. Week-16, @p < 0.05 IVAB Week-15 vs. Week-16, &p < 0.01 METO Week-15 vs. Week-16.
